# Occurrence of SARS-CoV-2 RNA in Six Municipal Wastewater Treatment Plants at the Early Stage of COVID-19 Pandemic in The United States

**DOI:** 10.3390/pathogens10070798

**Published:** 2021-06-23

**Authors:** Samendra P. Sherchan, Shalina Shahin, Jeenal Patel, Lauren M. Ward, Sarmila Tandukar, Sital Uprety, Bradley W. Schmitz, Warish Ahmed, Stuart Simpson, Pradip Gyawali

**Affiliations:** 1 Department of Environmental Health Sciences, Tulane University, 1440 Canal Street, Suite 2100, New Orleans, LA 70112, USA; sshahin@tulane.edu (S.S.); jpatel@tulane.edu (J.P.); lward9@tulane.edu (L.M.W.); 2 Policy Research Institute, Sano Gaucharan, Kathmandu 44600, Nepal; standukar@pri.gov.np or; 3 Swiss Federal Institute of Aquatic Science and Technology, Eawag, Überlandstrasse 133, Dübendorf, 8600 Zürich, Switzerland; sital.uprety@eawag.ch; 4 Yuma Center of Excellence for Desert Agriculture (YCEDA), University of Arizona, 6425 W. 8th St., Yuma, AZ 85364, USA; bschmitz@arizona.edu; 5 CSIRO Land and Water, Ecosciences Precinct, 41 Boggo Road, Dutton Park, QLD 4102, Australia; warish.ahmed@csiro.au; 6 CSIRO Land and Water, Lucas Heights, NSW 2234, Australia; Stuart.Simpson@csiro.au; 7 Institute of Environmental Science and Research Ltd., Porirua 5240, New Zealand; Pradip.Gyawali@esr.cri.nz

**Keywords:** COVID-19, SARS-CoV-2 RNA, wastewater, transmission risk, wastewater treatment

## Abstract

In this study, we investigated the occurrence of Severe Acute Respiratory Syndrome Coronavirus-2 (SARS-CoV-2) RNA in primary influent (*n* = 42), secondary effluent (*n* = 24) and tertiary treated effluent (*n* = 34) collected from six wastewater treatment plants (WWTPs A–F) in Virginia (WWTP A), Florida (WWTPs B, C, and D), and Georgia (WWTPs E and F) in the United States during April–July 2020. Of the 100 wastewater samples analyzed, eight (19%) untreated wastewater samples collected from the primary influents contained SARS-CoV-2 RNA as measured by reverse transcriptase quantitative polymerase chain reaction (RT-qPCR) assays. SARS-CoV-2 RNA were detected in influent wastewater samples collected from WWTP A (Virginia), WWTPs E and F (Georgia) and WWTP D (Florida). Secondary and tertiary effluent samples were not positive for SARS-CoV-2 RNA indicating the treatment processes in these WWTPs potentially removed SARS-CoV-2 RNA during the secondary and tertiary treatment processes. However, further studies are needed to understand the log removal values (LRVs) and transmission risks of SARS-CoV-2 RNA through analyzing wastewater samples from a wider range of WWTPs.

## 1. Introduction

Severe Acute Respiratory Syndrome Coronavirus-2 (SARS-CoV-2) is mainly transmitted through respiratory droplets; however, it has been detected in fecal samples and rectal swabs from infected people [1,2,3,4]. Viral shedding in the feces has been reported from day 1 to 33 after a negative nasopharyngeal swab and for up to 47 days after onset of COVID-19 symptoms [5]. Several recent studies have reported the presence of SARS-CoV-2 RNA in untreated wastewater in several countries [2,3,6,7,8,9,10]. Consequently, wastewater monitoring of SARS-CoV-2 RNA has been suggested as a non-invasive early warning tool and is currently being considered as a complementary tool for population-wide surveillance of COVID-19 pandemic [2,11,12].

Despite many reports of SARS-CoV-2 RNA detection in untreated municipal wastewater and primary sludge [2,3,4,6,7,8,9,13,14], data on presence in secondary treated and tertiary effluents are still limited [15]. Only a handful of studies have reported the occurrence of SARS-CoV-2 RNA in secondary treated effluent [8,11], while several studies could not detect SARS-CoV-2 RNA in treated effluents [3,8,9,10,16]. Haramoto and colleagues could not detect SARS-CoV-2 RNA in river water receiving chlorinated effluents [11], while Rimoldi et al. [10] detected SARS CoV-2 RNA in rivers receiving treated wastewaters in Italy during April 2020. Another study from Quito, Ecuador, reported the presence of SARS-CoV-2 RNA in river samples receiving untreated wastewaters [17]. 

Several papers suggested enteric transmission of SARS-CoV-2 can be possible via human waste and wastewater [18,19]. Therefore, it is important to determine the occurrence of SARS-CoV-2 RNA through wastewater treatment processes. The present study aimed to investigate the prevalence of SARS-CoV-2 RNA in untreated wastewater influents and treated effluents at six wastewater treatment plants (WWTPs) from three states (Virginia, Florida, and Georgia) in the United States in the early stage of the pandemic.

## 2. Material and Methods

### 2.1. Wastewater Sample Collection

Wastewater samples consisting of primary influent (*n* = 42), secondary-treated effluent (*n* = 24), and final tertiary effluent (*n* = 34) were collected between 28 April and 8 July 2020 from six WWTPs. These WWTPs are located in Virginia (WWTP A), Florida (WWTPs B, C, D), and Georgia (WWTPs E and F). Information about the treatment plant and the population served are provided by each respective water utility as demonstrated in Table 1. Composite wastewater samples (1L) were collected via 24-h autosamplers from influent and treated effluents in sterile Nalgene bottle bi-weekly during the study period. Sample bottles were kept in a cooler containing ice and shipped overnight to the laboratory at Tulane University. Upon arrival in the laboratory, samples were stored at −80 °C for two weeks due to delay in delivery of consumables.

### 2.2. SARS-CoV-2 Concentration

Untreated and treated wastewater samples were concentrated using three different techniques: ultrafiltration [7]; adsorption-elution [11]; and adsorption-extraction [20]. Remaining samples from April to July were processed using the adsorption-extraction method due to a shortage of supplies related to other methods. Ultrafiltration (Method A) applied the Centricon^®^ Plus-70 centrifugal filter with a nominal molecular weight limit (NMWL) of 100 kDa (Merck Millipore; part no UFC710008, Burlington, MA, USA). The adsorption-elution method (Method B) used an electronegative membrane as described elsewhere [3,21]. The adsorption-extraction method (Method C) also used an electronegative membrane, as well as a wastewater sample amended with MgCl_2_ pretreatment as reported previously [20].

### 2.3. Sample Process Control

Following virus concentration, 200 µL of the concentrated samples from Methods A and B, respectively, were then seeded with *Pseudomonas* bacteriophage Φ6 (DSM 21518). Φ6 addition acted as a sample process control (SPC) to determine the RNA extraction efficiency and identify the potential Reverse Transcriptase Quantitative Polymerase Chain Reaction (RT-qPCR) inhibition. Briefly, 2 μL of *Pseudomonas* bacteriophage Φ6 (2.0 × 10^5^ copies/μL) was seeded into 200 μL of concentrated wastewater samples and molecular biology grade water was used as a non-inhibitory control benchmark. The extraction efficiency (E) was calculated using the following equation

E = C/C_0_ × 100.

where C represents the observed Φ6-cDNA copy numbers per qPCR reaction in a wastewater sample, and C_o_ represents copy numbers per qPCR reaction in the control benchmark. RT-qPCR inhibition was determined by testing neat and 10-fold diluted RNA samples for Φ6. The differences between the neat and 10-fold diluted RNA for Φ6 were ≥ 3 *C_T_* values, and therefore considered to be potentially inhibitor free.

### 2.4. RNA Extraction and cDNA Preparation

Viral RNA was extracted from the concentrated samples and electronegative filter (200 μL) using a ZR Viral DNA/RNA Kit (Zymo Research, Irvine, CA, USA) to obtain a final volume of 100 µL, according to the manufacturer’s protocol. cDNA was prepared from 15 μL RNA using a High-Capacity cDNA Reverse Transcription Kit (Applied Biosystems, Foster City, CA, USA) according to the manufacturer’s instructions. The RT reaction mixture was incubated at 25 °C for 10 min, followed by 37 °C for 120 min, and a final incubation at 85 °C for 5 min to inactivate the enzyme.

### 2.5. RT-qPCR for SARS-CoV-2

RT-qPCR assays for SARS-CoV-2 was performed in BioRad CFX96 Real-Time PCR Instrument (BioRad Laboratories, Hercules, CA, USA) using CDC N1 and N2 primers and probes [3]. Reaction mixtures (25 μL) consisted of 12.5 μL of PerfecTa qPCR ToughMix (Quantabio, Beverly, MA, USA), 0.1 μL each of 100 μM forward and reverse primers, 0.05 μL of 100 μM TaqMan probe, and 2.5 μL of cDNA template. Details of PCR conditions and primer/probe information are provided in Table 2. The standard curve was generated using serial ten-fold dilution of the standard plasmid of SARS-CoV-2 and gBlock of Φ6 obtained from IDT (Coralville, IA, USA) [3]. Negative controls (DNase and RNase free water) were included to detect false-positive PCR amplification due to potential cross contamination. All RT-qPCR reactions were performed in duplicate. The sample was considered positive when both tubes fluoresced with sufficient intensity and the average cycle threshold (*C_T_*) value < 40 [22]. The slope of the standards ranged between −3.01 (CDC N2) to −3.34 (Φ6). Y-intercept values were −41 (Φ6), −39.17 (CDC N1), and −38.49 (CDC N2). The correlation coefficient (*R*^2^) values for these assays were 0.996% (CDC N1), 0.991% (CDC N2), and 0.999% (Φ6), respectively [3].

## 3. Results and Discussion

Several studies have reported the occurrence of SARS-CoV-2 RNA in untreated wastewater [2,3,6,7,8]. However, information regarding the persistence of SARS-CoV-2 and fate of its nucleic acid (RNA) in various stages of wastewater treatment processes is limited [25]. Such information is particularly important for assessing the likelihood of SARS-CoV-2 environmental transmission risk (if any) to humans or wildlife. As summarized in Table 3, SARS-CoV-2 RNA was detected in only eight untreated wastewater (influent) samples. Several factors may have contributed to the lower number of positive detections, including sample volume and type, virus concentration method used and the sensitivity of the assay and detection system [26]. Moreover, concentration of SARS-CoV-2 in wastewater may vary depending on several factors, such as number of infected people in the catchment, the type of sewer system, dilution, stormwater intrusion, and sampling types and time [26].

The concentration of SARS-CoV-2 RNA in wastewater is lower than other common enteric viruses such as noroviruses and adenoviruses [1]. COVID-19 patients may also excrete variable numbers of SARS-CoV-2 depending on the severity of diseases [27]. Therefore, wastewater samples require concentration prior to RNA extraction and RT-qPCR analysis. In this study, we used three virus concentration methods as no single method has been identified as a gold standard for the concentration of SARS-CoV-2 from wastewater. Among the concentration methods used, the adsorption-extraction method (Method C) yielded a greater rate of positive detections. Ahmed et al. [20] reported a greater recovery of murine hepatitis virus (MHV) in wastewater using adsorption-extraction method compared to ultrafiltration. The adsorption-elution method (Method B) did not yield any positive detections. Ahmed et al. [28] reported that the viral adsorption-elution method resulted in underestimation of the concentrations of human adenovirus and polyomavirus in environmental waters.

Notably, the average concentration of SARS-CoV-2 in influent wastewater samples by Method A ranged between 3.63 and 4.27 log_10_ GC/L, which was greater than Method C (2.93–3.39 log_10_ GC/L). We did not determine the recovery of concentration methods, but we used Φ6 as an RNA extraction process control. The mean recovery efficiencies of Φ6 were 94, 72, and 73% for Methods A, B, and C, respectively, demonstrating minimal viral genome loss during the RNA extraction. However, the whole recovery of the workflow (i.e., concentration and extraction) could be much lower. Of the six WWTPs, WWTP A, located in Virginia, had greater detection rates of SARS-CoV-2 RNA in influent wastewater compared to other WWTP influents. This was attributed to the fact that this WWTP was sampled in April to May 2020, when the clinical cases of COVID-19 increased in Virginia [29].

Of the two primer sets tested in this study, CDC N2 resulted in a greater frequency of detection (8/8) than CDC N1 (6/8). However, the average concentration (3.52 log_10_ GC/L) of SARS-CoV-2 RNA in influent wastewater samples determined using CDC N1 was slightly greater than CDC N2 assay (3.33 log_10_ GC/L). In contrast to our study, Barra et al. [30] and Vogels et al. [31] found a higher positive ratio for CDC N1 compared to that of the CDC N2 assay. Therefore, further validation studies would be useful to determine which assay(s) are generally more sensitive. In this study, SARS-CoV-2 RNA could not be detected in secondary- and tertiary-treated effluent samples, suggesting wastewater treatment processes such as MBR followed by activated carbon (WWTP A), Bardenpho (B) and Modified Ludzack Ettinger (MLE) process (WWTP D), as well as UV (WWTPs A, C), ozonation (E-F) disinfection processes in the studied WWTPs effectively degraded or removed SARS-CoV-2 RNA to concentrations below the detection limit. Studies conducted by Randazzo et al. [8] and Haramoto et al. [11] reported the presence of SARS-CoV-2 in secondary-treated wastewater in Spain and Japan, respectively. Interestingly, Haramoto et al. [11] could not detect SARS-CoV-2 RNA in untreated wastewater. This could be due to the fact that a small volume (200 mL) of untreated wastewater was tested in Haramoto’s study compared to secondary treated (5000 mL) wastewater results in reduced detection sensitivity in untreated wastewater.

Study conducted by Kumar et al. [32] found no SARS-CoV-2 in treated effluent samples in India. Other studies have reported the presence of SARS-CoV-2 RNA in treated wastewater effluents [33] and in river water in Ecuador and Italy [10,17]. However, infections with SARS-CoV-2 from treated wastewater appears unlikely based on no virus RNA being detected in samples after treatment. Rimoldi et al. [10] found no infectious SARS-CoV-2 in wastewater and river water samples in Italy using cell culture. A study of SARS-CoV-2 seeded into municipal wastewater and dechlorinated tap water found that SARS-CoV-2 was not highly persistent in aquatic environments compared to other pathogens [34]. Altogether, these observations suggest that the transmission risk of SARS-CoV-2 via treated wastewater is potentially negligible and requires further research on the infectivity of SARS-CoV-2 in wastewater.

The present study has several limitations, and, therefore, the results presented need to be interpreted with care. First, samples were stored at −80 °C for two weeks due to lack of consumables, and this may have impacted our results. A study reported the potential degradation of SARS-CoV-2 RNA in wastewater [15]. However, Ahmed et al. [34] found the average *T*_90_ (time required for 1-log_10_ reduction) of SARS-CoV-2 RNA ranged from 8.04 to 27.8 days in untreated wastewater. Therefore, SARS-CoV-2 RNA can persist long enough in wastewater for accurate detection. Second, samples were collected from the various stages of treatment processes on the same day due to lack of logistics. It would be better to perform sampling on consecutive days based on retention time to determine the treatment efficacy. The volume of wastewater analyzed from secondary and primary effluent samples may have reduced the detection sensitivity. This study provides a snapshot of the prevalence of SARS-CoV-2 over several months. Further studies with a larger number of samples from different types of wastewater treatment plants would generate a robust dataset on the reduction values of SARS-CoV-2 through a range of WWTPs. Such information will enable facilities and researchers to determine the fate and transport of the virus throughout their treatment process and consider operations that may optimize treatment performance to minimizing virus transmission into the environment.

## 4. Conclusions

- Wastewater samples from influents of WWTPs in Virginia, Florida (WWTP D) and Georgia tested positive for SARS-CoV-2 RNA.
- SARS-CoV-2 RNA was detected in 19% (8/42) untreated wastewater influent samples and tested negative for all 24 secondary- and 34 tertiary-treated effluents.
- The prevalence of SARS-CoV-2 RNA was low in the studied WWTPs in the early pandemic stage.
- Both ultrafiltration and adsorption-extraction methods were effective for detecting RNA in wastewater samples.

## Figures and Tables

**Table 1:** Data on WWTPs in the Studied Areas.

WWTPs	Location ^a^	Population Served ^b^	Treatment Train
A	VA	300,000	preliminary screening, grit removal, primary clarification, fine screening, flow equalization, membrane bioreactors (MBR), activated carbon, and UV disinfection.
B/C	FL	974,996	WWTP B uses an advanced Bardenpho process while WWTP C uses UV disinfection
D	FL	471,826	a Modified Ludzack Ettinger (MLE) process configuration with two biological treatment trains.
E/F	GA	936,250	WWTP E uses advanced membrane bioreactor (MBR) wastewater treatment, WWTP F uses biological activated carbon (BAC) and ozonation

^a^ VA: Virginia, FL: Florida, GA: Georgia ^b^ provided by each respective water utility.

**Table 2:** Oligonucleotide sequences of primers and probes used in this study.

Assay	Target Gene	Primer/Probe	Sequence (5′ > 3′) ^a^	PCR Conditions	Reference
N1	Nucleocapsid (N)	2019-nCoV_N1-F	GAC CCC AAA ATC AGC GAA AT	95 °C for 10 min and 45 cycles of 95 °C for 10 s and 55 °C for 30 s	[23]
		2019-nCoV_N1-R	TCT GGT TAC TGC CAG TTG AAT CTG		
		2019-nCoV_N1-P	FAM-ACCCCGCATTACGTTTGGTGGACC-BHQ1		
N2	Nucleocapsid (N)	2019-nCoV_N2-F	TTA CAA ACA TTG GCC GCA AA	95 °C for 10 min and 45 cycles of 95 °C for 10 s and 55 °C for 30 s	[23]
		2019-nCoV_N2-R	GCG CGA CAT TCC GAA GAA		
phi6	phi-6S 1	2019-nCoV_N2-P phi6- F phi6- R phi6- P	FAM-ACAATTTGCCCCCAGCGCTTCAG-BHQ1 TGGCGGCGGTCAAGAGC GGATGATTCTCCAGAAGCTGCTG FAM/CGGTC GTCG/ZEN/CAGGTCTGACACTCGC/3IABkFQ/	94 °C for 3 min followed by 35 cycles of 94 °C for 15 s and 60 °C for 1 min	[24]

^a^ FAM, 6-carboxyfluorescein; BHQ1, black hole quencher 1.

**Table 3:** Prevalence of SARS-CoV-2 RNA in wastewater.

States	WWTPs	Sample	No of Samples Tested	No. of Positive (%)		GC/L	
				N1	N2	N1	N2
Virginia	A	Influent	10	(1/3) *^a^* (2/10) *^b^*	(1/3) *^a^* (2/10) *^b^*	4.3 × 10^3 *a*^ 3.36 × 10^3 *b*^ 3.43 ×10^3 *b*^	4.4 × 10^3 *a*^ 1.70 × 10^3 *b*^ 3.30 × 10^2 *b*^
		Effluent	12	(0/3) *^a^* (0/12) *^b^*	(0/3) *^a^* (0/12) *^b^*		
Florida	B	Influent	6	(0/2) *^a^* (0/6) *^b^*	(0/2) *^a^* (0/6) *^b^*		
		Secondary Effluent	4	(0/2) *^a^* (0/4) *^b^*	(0/2) *^a^* (0/4) *^b^*		
		Effluent	4	(0/1) *^a^* (0/4) *^b^*	(0/1) *^a^* (0/4) *^b^*		
	C	Influent	6	(0/2) *^a^* (0/6) *^b^*	(0/2) *^a^* (0/6) *^b^*		
		Secondary Effluent	4	(0/2) *^a^* (0/4) *^b^*	(0/2) *^a^* (0/4) *^b^*		
		Effluent	4	(0/2) *^a^* (0/4) *^b^*	(0/2) *^a^* (0/4) *^b^*		
	D	Influent	10	(1/10) *^b^*	(2/10) *^b^*	8.70 × 10^2 *b*^	8.0 × 10^2 *b*^ 9.3 × 10^2 *b*^
		Secondary Effluent	6	(0/6) *^b^*	(0/6) *^b^*		
		Effluent	4	(0/6) *^b^*	(0/6) *^b^*		
Georgia	E	Influent	6	(1/2) *^a^* (1/6) *^b^*	(1/2) *^a^* (1/6) *^b^*	1.0 × 10^4 *a*^ 1.5 ×10^3 *b*^	7.8 × 10^3 *a*^ 1.70 × 10^3 *b*^
		Secondary Effluent	6	(0/1) *^a^* (0/6) *^b^*	(0/1) *^a^* (0/6) *^b^*		
		Effluent	6	(0/1) *^a^* (0/6) *^b^*	(0/1) *^a^* (0/6) *^b^*		
	F	Influent	4	(1/2) *^a^* (0/4) *^b^*	(1/2) *^a^* (0/4) *^b^*	6.5 × 10^3 *b*^	1.9 × 10^4 *b*^
		Secondary Effluent	4	(0/2) *^a^* (0/4) *^b^*	(0/2) *^a^* (0/4) *^b^*		
		Effluent	4	(0/2) *^a^* (0/4) *^b^*	(0/2) *^a^* (0/4) *^b^*		

*^a^* Ultrafiltration method, *^b^* Adsorption-extraction.

## Data Availability

Available upon request.
